# Psychosocial Variables Associated with Immunosuppressive Medication Non-Adherence after Renal Transplantation

**DOI:** 10.3389/fpsyt.2018.00023

**Published:** 2018-02-15

**Authors:** Jennifer Felicia Scheel, Katharina Schieber, Sandra Reber, Lisa Stoessel, Elisabeth Waldmann, Sabine Jank, Kai-Uwe Eckardt, Franziska Grundmann, Frank Vitinius, Martina de Zwaan, Anna Bertram, Yesim Erim

**Affiliations:** ^1^Department of Psychosomatic Medicine and Psychotherapy, University Hospital Erlangen, Friedrich-Alexander University Erlangen-Nürnberg (FAU), Erlangen, Germany; ^2^Department of Medical Informatics, Biometry and Epidemiology, Friedrich-Alexander University Erlangen-Nürnberg, Erlangen, Germany; ^3^Department of Nephrology and Hypertension, University Hospital Erlangen, Friedrich-Alexander University Erlangen-Nürnberg (FAU), Erlangen, Germany; ^4^Department II of Internal Medicine, Nephrology, Rheumatology, Diabetes and General Internal Medicine, University Hospital of Cologne, Cologne, Germany; ^5^Department of Psychosomatic Medicine and Psychotherapy, University Hospital of Cologne, Cologne, Germany; ^6^Department of Psychosomatic Medicine and Psychotherapy, Hannover Medical School, Hannover, Germany; ^7^Department of Nephrology and Hypertension, Hannover Medical School, Hannover, Germany

**Keywords:** renal transplantation, immunosuppressive medication non-adherence, social support, depression, anxiety, quality of life

## Abstract

**Introduction:**

Non-adherence to immunosuppressive medication is regarded as an important factor for graft rejection and loss after successful renal transplantation. Yet, results on prevalence and relationship with psychosocial parameters are heterogeneous. The main aim of this study was to investigate the association of immunosuppressive medication non-adherence and psychosocial factors.

**Methods:**

In 330 adult renal transplant recipients (≥12 months posttransplantation), health-related quality of life, depression, anxiety, social support, and subjective medication experiences were assessed, and their associations with patient-reported non-adherence was evaluated.

**Results:**

33.6% of the patients admitted to be partially non-adherent. Non-adherence was associated with younger age, poorer social support, lower mental, but higher physical health-related quality of life. There was no association with depression and anxiety. However, high proportions of clinically relevant depression and anxiety symptoms were apparent in both adherent and non-adherent patients.

**Conclusion:**

In the posttransplant follow-up, kidney recipients with lower perceived social support, lower mental and higher physical health-related quality of life, and younger age can be regarded as a risk group for immunosuppressive medication non-adherence. In follow-up contacts with kidney transplant patients, physicians may pay attention to these factors. Furthermore, psychosocial interventions to optimize immunosuppressive medication adherence can be designed on the basis of this information, especially including subjectively perceived physical health-related quality of life and fostering social support seems to be of importance.

## Introduction

After successful renal transplantation, graft survival is of utmost importance and non-adherence to immunosuppressive medication is regarded as an important factor for graft rejection and graft loss (1–5). The prevalence of non-adherence in renal transplant recipients varies between 2.4 and 78.0% in different studies (4–10). It is crucial to understand the factors that promote better adherence, to identify kidney recipients with insufficient immunosuppressive medication adherence in the posttransplant follow-up, and to support them with professional psychosocial interventions. About 40% of kidney transplant recipients are interested in a support group (11). There is first evidence that adherence after solid-organ transplantation can be improved by psychosocial interventions, such as educational, behavioral, and counseling interventions (12–18). Psychological factors, which might promote onset and maintenance of immunosuppressant medication adherence, can be objectives of psychotherapeutic interventions. In their review, Low et al. (12) state that interventions that focus on behavioral risk factors or a combination of behavioral, educational, and emotional factors are effective in promoting adherence. Furthermore, the participation of the patients should be encouraged (12). Also, De Bleser et al. (13) concluded that combining interventions in a team approach might be effective for the management of a chronic disease like organ transplant patients have. To improve psychosocial interventions and tailor them to risk patients, we need more information on psychosocial factors that are highly associated with non-adherence.

Until today, non-adherence after renal organ transplantation has been shown to be associated with anxiety (1, 3, 8), poor social support (1, 3, 19–23), poor quality of life (7, 10, 24), attitudes and beliefs regarding immunosuppressive medication (7–9, 25–27), and depressive symptoms (1, 3, 6, 8, 25, 28–34), which are present in approximately 25% of renal transplant recipients (28). Yet, there are also studies with non-significant results regarding non-adherence and depression (25, 27, 30), anxiety (30), attitudes and beliefs regarding immunosuppressive medication (35, 36), and social support (27, 30). In their present meta-analysis (across different transplantation types), Ladin et al. (37) state that social support is only inconsistently and weakly associated with non-adherence.

Hence, not only the prevalence rates of patient-reported non-adherence but also the (strengths of) associations with psychosocial parameters are heterogeneous. This might be due to different ways to assess non-adherence (self-report or expert-report, pill count, electronic monitoring, immunosuppressant through levels), different tools to assess potential psychosocial correlates of non-adherence, and different socioeconomic and cultural conditions in the transplant patient samples. The studies mentioned above were conducted in different countries (mainly in the United States) with different health care systems and legal frameworks, which might influence the pretransplant evaluation and also the posttransplant non-adherence behavior (e.g., due to the varying costs of immunosuppressive medication or the varying psychosocial criteria for receiving an organ transplantation). Especially in the light of the ongoing debate regarding reproducibility of scientific findings [e.g., Heino et al. (38)], replication is required. Furthermore, the association of non-adherence and quality of life has not extensively been investigated although quality of life is increasingly perceived as an additional core outcome beside (graft) survival after successful transplantation (39).

Therefore, the aim of this study is to investigate the associations of patient-reported non-adherence with psychosocial factors, which could be targeted in psychosocial interventions. We hypothesized that there would be a significant association between non-adherence and high levels of depression, anxiety, and negative beliefs regarding immunosuppressive medication, low social support, and low health-related quality of life.

## Materials and Methods

### Patients

This cross-sectional multicenter study was performed at the transplant centers of three German University Hospitals and included 330 consecutive adult patients, who presented at the transplant center for follow-up examinations between November 2014 and December 2015 and agreed to take part in the study. They had undergone renal transplantation between 1981 and 2014; exclusion criteria encompassed being <1 year posttransplantation, additional non-renal transplantation, mental disability, and insufficient German language skills for understanding of the questionnaires.

Institutional ethics committee approval was obtained in each participating institution and has been performed in accordance with the ethical standards as laid down in the 1964 Declaration of Helsinki and its later amendments or comparable ethical standards. All participants gave written informed consent.

### Patient-Reported Immunosuppressive Medication Non-Adherence

For the assessment of patient-reported immunosuppressive medication non-adherence, the Basel Assessment of Adherence to Immunosuppressive Medications Scale [BAASIS© (40); German version by the Leuven-Basel Adherence Research Group (41), self-report instrument; Cronbach’s alpha in our sample 0.39] was used. It consists of four items (dose taking, drug holidays, timing deviation >2 h from prescribed time, dose reduction) rated on a six-point scale (0 = never, 5 = every day). Non-adherence was defined as at least one affirmative answer to any of the four items (dichotomous score). Following a systematic review of self-report instruments to identify medication non-adherence, the BAASIS© is recommended as a reliable, valid, and sensitive tool (40).

### Psychosocial Variables Potentially Associated with Patient-Reported Non-Adherence

#### Depression and Anxiety

Depression and anxiety were measured with the HADS (42), an internationally widely used reliable and valid self-report instrument for the assessment of anxiety and depression among medically ill patients (43) (German version by Herrmann et al. (42); Cronbach’s alpha in our sample 0.83 for anxiety and 0.79 for depression). Each of the two scales consists of seven items (sum score ranging from 0 to 21). Higher scores indicate higher levels of depressive or anxiety-related symptoms. Scores lower than 7 are considered as clinically not relevant, scores from 8 to 10 are considered “borderline”, and scores >10 are indicative for clinically relevant depression and anxiety.

#### Perceived Social Support

Social support (practical support, emotional support, and social integration) was assessed with the seven-item short form of the German Social Support Questionnaire [F-SozU (44, 45); self-report instrument; Cronbach’s alpha in our sample 0.92]. Total scores range from 7 to 35; higher scores are indicative of higher perceived social support.

#### Perceived Health-Related Quality of Life

Health-related quality of life was measured by the widely used 12-item short version of the Short Form Health Survey SF-36 [SF-12 (46); German version by Bullinger and Kirchberger (47) and validation by Gandek et al. (48); self-report instrument; Cronbach’s alpha in our sample 0.60 for physical health-related quality of life and 0.58 for mental health-related quality of life]. It consists of the two subscales “Physical Component Summary” and “Mental Component Summary,” which both range from 0 to 100. Higher scores indicate higher subjective health-related quality of life.

#### Subjective Experiences and Attitudes toward Immunosuppressant Medications

The German Medication Experience Scale for Immunosuppressants (MESI) (49) is a seven-item self-report questionnaire that assesses subjective experiences and attitudes toward immunosuppressive medication (Cronbach’s alpha in our sample 0.77). The total score ranges from 4 to 33; higher values indicate more negative experiences and attitudes.

### Statistical Analyses

In the case of missing variables (two patients did not answer the HADS), the patients were excluded from the respective analyses. For an overview, means, SDs, medians, ranges, and frequencies (as appropriate) are given of all variables. For the analysis of group differences, *t*-tests [normally distributed continuous variables; effect size measure: *d*; *d* ≥ 0.2 small, *d* ≥ 0.5 medium, *d* ≥ 0.8 large effect size (50)], Mann–Whitney *U*-tests [not normally distributed continuous variables; effect size measure: *r*; *r* ≥ 0.1 small, *r* ≥ 0.3 medium, *r* ≥ 0.5 large effect size (50)], and χ^2^ tests [dichotomous variables; effect size measure: Phi; Phi > 0.1 small, Phi > 0.3 medium, Phi > 0.5 large effect size (50)] were calculated. In addition, a logistic regression model was conducted with the variables significantly differing between adherent and non-adherent patients as independent variables and self-reported non-adherence as the dependent variable. For all analyses, the statistical analysis program SPSS 21 was used. Findings were considered to be statistically significant at α < 0.05.

## Results

330 patients (65.2% male and 34.8% female; mean age, 52.9 ± 13.8 years at assessment) participated. According to the BAASIS, 33.6% of the patients reported non-adherence. Detailed information concerning sociodemographic and medical variables for both adherent and non-adherent patients is presented in Table 1. Adherent and non-adherent patients differed significantly concerning current age and age at the time of transplantation, but not concerning time since transplantation, sex, dialysis before transplantation, number of renal transplantations, and type of renal graft.

**Table 1:** Group differences between adherent and non-adherent patients concerning sociodemographic and medical variables.

	Total sample	Adherent patients	Non-adherent patients	Statistics for difference between groups		
				*U*, *t*, χ^2^	*p*	Effect size
Age (years)						
Mean (SD)	52.9 (13.8)	54.7 (13.5)	49.3 (13.6)	*U* = 9460.0	**0.001**	0.20 (*r*)
Median (IQR)	54.0 (43.0–64.0)	56.0 (46.0–65.0)	51.0 (41.0–58.0)			
Range	18.0–80.0	19.0–80.0	18.0–75.0			
*n*	330	219	111			

Age at the time of transplantation (years)						
Mean (SD)	46.1 (14.6)	47.9 (14.7)	42.4 (13.8)	*t*(328) = −3.247	**0.001**	0.38 (*d*)
Range	2–76	2–76	10.0–71.0			
*n*	330	219	111			

Time since last transplantation (years)						
Mean (SD)	6.8 (6.2)	6.8 (6.5)	6.9 (5.7)	*U* = 11625.0	0.516	0.04 (*r*)
Median (IQR)	4.0 (3.0–9.0)	4.0 (3.0–9.0)	5.0 (3.0–9.0)			
Range	1.0–34.0	1.0–34.0	1.0–26.0			
*n*	330	219	111			

Sex						
Female (%)	115 (34.8)	78 (35.6)	37 (33.3)	χ^2^ (1, *n* = 330) = 0.169	0.681	−0.02 (Phi)
Male (%)	215 (65.2)	141 (64.4)	74 (66.7)			
*n*	330	219	111			

Dialysis before transplantation						
Yes (%)	293 (89.3)	194 (89.4)	99 (89.2)	χ^2^ (1, *n* = 328) = 0.003	0.953	<0.01 (Phi)
No (%)	35 (10.7)	23 (10.6)	12 (10.8)			
*n*	328	217	111			

>1 renal transplantation						
Yes (%)	38 (11.5)	30 (13.7)	8 (7.2)	χ^2^ (1, *n* = 330) = 3.046	0.081	0.10 (Phi)
No (%)	292 (88.5)	189 (86.3)	103 (92.8)			
*n*	330	219	111			

Type of renal graft						
Postmortem (%)	223 (67.6)	152 (69.4)	71 (64.0)	χ^2^ (1, *n* = 330) = 0.996	0.318	0.06 (Phi)
Living (%)	107 (32.4)	67 (30.6)	40 (36.0)			
*n*	330	219	111			

*Significant values are marked in bold*

With regard to psychosocial functioning, adherent and non-adherent patients differed significantly concerning social support and physical and mental health-related quality of life, but not concerning depression, anxiety, and negative beliefs regarding immunosuppressive medication. For details, please see Table 2. Generally, 16.7% of the patients in this study exhibited clinically relevant depressive symptoms (HADS depression score, >10) and 63.9% clinically relevant anxiety symptoms (HADS anxiety score, >10). However, the adherent and the non-adherent patient groups did not differ significantly regarding the proportion of patients with clinically relevant depressive symptoms (16.6% of the adherent patients and 17.1% of the non-adherent patients) and clinically relevant anxiety symptoms (62.7% of the adherent patients and 67.6% of the non-adherent patients). Overall, effect sizes for the differences were small (Table 2).

**Table 2:** Group differences between adherent and non-adherent patients concerning psychosocial variables.

	Total sample	Adherent patients	Non-adherent patients	Statistics for difference between groups		
				*U*, *t*, χ^2^	*p*	Effect size
Depression continuous (HADS)						
Mean (SD)	7.9 (3.3)	7.7 (3.4)	8.4 (2.9)	*U* = 34940.0	0.347	0.06 (*r*)
Median (IQR)	9.0 (7.0–10.0)	9.0 (6–10)	9.0 (7.0–10.0)			
Range	0.0–19.0	0.0–19.0	0.0–18.0			
*n*	328	217	111			

Depression dichotomous (HADS; low: score ≤ 10; high/clinically relevant: score > 10)						
Low (%)	273 (83.3)	181 (83.4)	92 (82.9)	χ^2^ (1, *n* = 328) = 0.015	0.904	0.01 (Phi)
High (%)	55 (16.7)	36 (16.6)	19 (17.1)			
*n*	328	217	111			

Anxiety continuous (HADS)						
Mean (SD)	10.8 (4.3)	10.4 (4.6)	11.4 (3.5)	*U* = 35084.0	0.449	0.05 (*r*)
Median (IQR)	12.0 (8.3–14.0)	12.0 (7–14)	12.0 (10.0–14.0)			
Range	0.0–21.0	0.0–16.0	2.0–21.0			
*n*	328	217	111			

Anxiety dichotomous (HADS; low: score ≤ 10; high/clinically relevant: score > 10)						
Low (%)	117 (36.1)	81 (37.3)	36 (32.4)	χ^2^ (1, *n* = 328) = 0.767	0.381	0.05 (Phi)
High (%)	211 (63.9)	136 (62.7)	75 (67.6)			
*n*	328	217	111			

Social support (FSozU)						
Mean (SD)	29.1 (7.1)	29.8 (6.9)	27.8 (7.5)	*U* = 16082.0	**0.004**	0.17 (*r*)
Median (IQR)	31.5 (26.0–35.0)	33.0 (27.0–35.0)	30.0 (23.0–34.0)			
Range	70–35.0	7–35.0	7.0–35.0			
*n*	330	219	111			

Physical component score health-related quality of life (SF-12)						
Mean (SD)	44.9 (10.7)	44.0 ± 10.8	46.8 ± 10.4	*U* = 34184.0	**0.012**	0.15 (*r*)
Median (IQR)	47.8 (36.8–54.2)	46.5 (36.4–53.8)	50.4 (38.4–55.3)			
Range	15.7–67.7	15.7–67.7	18.7–61.8			
*n*	330	219	111			

Mental component score health-related quality of life (SF-12)						
Mean (SD)	48.9 (10.3)	49.7 ± 10.0	47.3 ± 10.8	*U* = 16604.0	**0.031**	0.13 (*r*)
Median (IQR)	52.2 (41.9–57.1)	52.8 (43.4–57.8)	50.2 (39.7–55.9)			
Range	19.6–66.5	23.0–66.5	19.6–62.4			
*n*	330	219	111			

Subjective experience of IS medication (Medication Experience Scale for Immunosuppressants)						
Mean (SD)	15.1 (5.9)	14.8 ± 6.1	15.6 ± 5.7	*U* = 111460.0	0.395	0.10 (r)
Median (IQR)	16 (12–19.0)	16.0 (10–19)	16.0 (12–19)			
Range	4–28	4–28	4–26			
*n*	330	219	111			

*Significant values are marked in bold*

When including the variables significantly differing between adherent and non-adherent patients in a logistic regression model, patient-reported non-adherence could be predicted significantly (9.0% explained variance, *p* > 0.001). Age at the time of transplantation and physical health-related quality of life proved to be significant single predictors, social support was in trend significant, and mental health-related quality of life failed to reach significance. For details, please see Table 3.

**Table 3:** Logistic regression model for the prediction of self-reported non-adherence.

	Regression coefficient B (SE)	Wald	Odds ratio	95% confidence interval	*p* (single predictors)	Explained variance (Nagelkerke’s*R*^2^) whole model	*p* (whole model)	−2 Log-likelihood
Age at the time of transplantation	−0.022	6.880	0.978	0.962–0.994	0.009	0.090	<0.001	399.333
Social support (FSozU)	−0.035	3.804	0.966	0.933–1.000	0.051			
Physical component score health-related quality of life (SF-12)	0.030	6.053	1.030	1.006–1.055	0.014			
Mental component score health-related quality of life (SF-12)	−0.015	1.352	0.985	0.961–1.010	0.245			

### Gender Analyses

Male and female patients differed significantly only regarding age. Female patients were younger at the time of assessment (49.7 years versus 54.6 years in males) and at the time of transplantation (43.3 years versus 47.6 years in males). When comparing non-adherent male and female patients, no differences were found regarding mental and physical health-related quality of life, perceived social support, anxiety, depression, and age. When comparing adherent and non-adherent patients in the male subsample, the same pattern as in the total sample emerged: non-adherent male patients exhibited lower mental and higher physical health-related quality of life, lower social support, and were younger both at assessment and at the time of transplantation. When comparing adherent and non-adherent patients in the female subsample, the pattern differed from the total sample as no significant differences emerged. Yet, physical health-related quality of life was in trend higher (*p* = *0.078*) and social support in trend lower (*p* = *0.069*) in non-adherent female patients.

## Discussion

The aim of this study is to investigate how patient-reported immunosuppressive medication non-adherence is associated with psychosocial factors. About one-third of the patients in the present sample admitted to be non-adherent. Patient-reported non-adherence was associated with poorer social support and poorer mental, but better physical health-related quality of life. Furthermore, non-adherence was related to younger age, but not to time since transplantation.

The negative relationship between non-adherence and perceived social support is in line with previous research, which indicates associations of poor social support and non-adherence (1, 3, 19–23). Chisholm-Burns et al. (20) concluded that social support might “buffer” the negative impact of stress, help to effectively manage stress, and, thus, might positively influence adherence behavior. Ladin et al. (37) stated in their meta-analysis (across different transplantation types) that social support is only inconsistently and weakly associated with non-adherence. Yet, they reported social support (measured by marital status) to vary across different transplantation types and to be related to non-adherence, particularly in kidney transplant recipients. Furthermore, they found differences between received and subjectively perceived social support: higher levels of *perceived* social support were significantly related to higher medication adherence (37).

Although the association of non-adherence with lower mental health-related quality of life is in line with our hypotheses and the literature (7), its correlation with higher physical health-related quality of life is opposite to our expectations. Putatively, physical health-related quality of life could be associated with worse adherence behavior because patients with higher physical health-related quality of life might have a more mobile life style resulting in more perceived behavioral barriers regarding adherence (e.g., more traveling) and might perceive a lower threat of disease, which could lead to worse adherence behavior according to the health belief model (51).

Contrary to our expectations, we did not find associations between non-adherence and both depression and anxiety, although several studies suggest associations between non-adherence and depression (1, 3, 6, 8, 25, 28–34) as well as non-adherence and anxiety (1, 3, 8). In this study, 63.9% of the patients exhibited clinically relevant anxiety symptoms (HADS anxiety score, >10), which is much higher than the proportion found in the study of Weng et al. (30) (6.0% with HADS anxiety score, >10). Furthermore, 16.7% of the patients in this study exhibited clinically relevant depressive symptoms. The prevalence of clinically relevant depressive symptoms (HADS depression score, >10) is comparable to the findings of Chilcot et al. (28) (approximately 25% according to the Beck Depression Inventory) and Griva et al. (25) (13.2% according to the Beck Depression Inventory), but remarkably higher than the findings reported by Weng et al. (30) (4.8% with a HADS depression score, ≥8 and 2.0% with a HADS depression score, >10). Yet, Weng et al. (30) could not show significant associations between non-adherence (*via* immunosuppressant through level variability) and depression after adjusting for sociodemographic variables (age, income, and employment status). Also, forgetfulness and intentional omission have to be taken into account regarding the association of depression and patient-reported non-adherence, as Griva et al. (25) found depression to be only associated with *intentional* non-adherence, but not with unintentional non-adherence due to forgetfulness.

Also contrary to our expectations, we did not find differences between adherent and non-adherent patients regarding subjective (negative) experiences and attitudes toward immunosuppressant medications (MESI) like we did in the previous study of our research group (8). This is surprising but might be due to generally high scores regarding negative subjective experiences and attitudes toward immunosuppressant medications (mean of the group > cut-off 15) and a high percentage of patients with scores > cut-off 15 (58.2%) in this study.

When analyzing the data separately for male and female patients, similar results as in the total sample emerged, but significance was reduced for male patients and mostly disappeared for female patients (although the same patterns of differences could be seen descriptively). In general, males are overrepresented in transplant patient samples. Females are less likely to be included on the transplant waiting list than men (52–55) and also less likely to receive a transplant than males (gender bias in treatment) (53, 55). Furthermore, two-thirds of the organ donors, but only one-third of the organ recipients are female (accounts for different age and ethnic groups) (56). In line, we found a 2 to 1 male-to-female ratio, which was also the case in the study by Goetzmann et al. (57). Further research should comprise even larger samples to be able to detect potential gender differences in the comparison of adherent and non-adherent patients.

The current literature indicates that non-adherence can be improved by psychosocial interventions, such as educational, behavioral, and counseling interventions, but these effects are often not satisfactory (12–15). This might on the one hand be due to small sample sizes, but on the other hand could be caused by not sufficiently considering psychosocial correlates of non-adherence when developing these interventions. Taking our results into account, a particular focus in an intervention could be to increase the awareness of the importance of immunosuppressive medication adherence, especially in patients who perceive physical health-related quality of life (still) as sufficient and might therefore be tempted to non-adherence. A second focus could be improving social support, as social support might be an important “buffer” against the negative impact of stress and/or an effective help regarding stress management and this might positively influence adherence behavior (20). Social support might also be utilized to improve the management of complex medication regimens and tackle forgetfulness (20). However, our results have to be interpreted with caution as the effect sizes in univariate analyses were small, and in the multivariate regression model, there was only a trend for a negative association between perceived social support and self-reported non-adherence.

Future studies should also examine the impact of social support with the psychodynamic theories of attachment. Attachment theory describes that people with secure attachment promote adaptive responses to threat throughout the lifespan (58). Hence, attachment style could contribute to explain the crucial role of social support in adherence. Calia et al. (59) found that attachment style and adherence were interrelated in kidney recipients. In a more psychodynamic approach, attachment theory aspects, resistance (to treatment), adaptive and maladaptive defense mechanisms in disease coping, and (counter)transference could be therapeutically approached (60). Moreover, Mintz (61) stated that a psychodynamic psychopharmacology is required, because meaning and interpersonal factors should be addressed explicitly in pharmacological treatment. Patients might have conscious or unconscious goals, fears etc., which might interfere with the desired medication effects and could lead to both non-adherence and/or the repeated experience of adverse effects of the medication (61).

At least, the association of non-adherence and age has to be considered: non-adherent patients were significantly younger than adherent patients at the time of assessment and also at the time of transplantation, whereas time since transplantation did not differ significantly between adherent and non-adherent patients. These findings are similar to those of Butler et al. (62). Younger adult age at assessment and at the time of transplantation might be associated with poorer patient-reported adherence due to a lower perceived threat of disease or probability of health problems in younger years. For example, younger adults estimate their risk to experience health hazards like heart disease to be lower than older adults in the general population (63).

There are two limitations that need to be considered. First, the retrospective, cross-sectional design does not allow causal conclusions regarding the associations of patient-reported non-adherence and psychosocial variables. Second, the possibility of socially desirable answers must be considered in patients contacted during their scheduled visits at the transplantation center. As patient-reported immunosuppressive medication non-adherence varies widely, the gold standard for measuring non-adherence would have been electronic medication monitoring systems, as they are a more objective reference method for non-adherence. Yet, these systems are impractical for clinical routine and very expensive. As non-adherence after renal organ transplantation has also been shown to be associated with other factors than the ones investigated in this study, e.g., personality traits like “openness” (6), these variables should be further investigated in future studies.

## Conclusion

In the posttransplant follow-up, kidney recipients with lower perceived social support, lower mental and higher physical health-related quality of life, and younger age can be regarded as a risk group for immunosuppressive medication non-adherence. Depression and anxiety seem to be unrelated to non-adherence. Physicians may pay attention to these factors in follow-up contacts with kidney transplant patients. Moreover, psychosocial interventions for the optimization of immunosuppressive medication adherence can be designed on the basis of this information. Particularly fostering social support and including subjectively perceived physical health-related quality of life and its behavioral consequences seems to be of importance.

## Ethics Statement

Institutional ethics committee approval was obtained in each participating institution [Ethics Committee of the University Hospital Erlangen, Friedrich-Alexander University Erlangen-Nürnberg (FAU), Ethics Committee of the Hannover Medical School, Ethics Committee of the University Hospital of Cologne] and has been performed in accordance with the ethical standards as laid down in the 1964 Declaration of Helsinki and its later amendments or comparable ethical standards. All participants gave written informed consent.

## Author Contributions

Substantial contributions to the conception or design of the work: JS, FV, MZ, and YE. Substantial contributions to the acquisition, analysis, or interpretation of data for the work: JS, KS, SR, LS, EW, SJ, K-UE, FG, FV, MZ, AB, and YE. Drafting the work or revising it critically for important intellectual content: JS, KS, SR, LS, EW, SJ, K-UE, FG, FV, MZ, AB, and YE. Final approval of the version to be published: JS, KS, SR, LS, EW, SJ, K-UE, FG, FV, MZ, AB, and YE. Agreement to be accountable for all aspects of the work in ensuring that questions related to the accuracy or integrity of any part of the work are appropriately investigated and resolved: JS, KS, SR, LS, EW, SJ, K-UE, FG, FV, MD, AB, and YE. Patient recruitment in Erlangen was performed by SR and LS in fulfillment of the requirements for obtaining the degree “Dr. med.”: MD.

## Conflict of Interest Statement

The authors declare that the research was conducted in the absence of any commercial or financial relationships that could be construed as a potential conflict of interest.
